# The interplay of inflammation and remodeling in the pathogenesis of chronic rhinosinusitis: current understanding and future directions

**DOI:** 10.3389/fimmu.2023.1238673

**Published:** 2023-09-12

**Authors:** Xinru Gong, Zhoutong Han, Hongli Fan, Yuqi Wu, Yuanqiong He, Yijie Fu, Tianmin Zhu, Hui Li

**Affiliations:** ^1^ Health and Rehabilitation College, Chengdu University of Traditional Chinese Medicine, Chengdu, Sichuan, China; ^2^ School of Preclinical Medicine, Chengdu University, Chengdu, China

**Keywords:** CRS, remodeling, inflammation, TGF-β, TNF-α, neutrophils

## Abstract

Chronic rhinosinusitis (CRS), a common clinical condition characterized by persistent mucosal inflammation and tissue remodeling, has a complex pathogenesis that is intricately linked to innate and adaptive immunity. A number of studies have demonstrated that a variety of immune cells and cytokines that play a vital role in mediating inflammation in CRS are also involved in remodeling of the nasal mucosa and the cells as well as different cytokines involved in remodeling in CRS are also able to exert some influence on inflammation, even though the exact relationship between inflammation and remodeling in CRS has not yet been fully elucidated. In this review, the potential role of immune cells and cytokines in regulating inflammation and remodeling of CRS mucosa has been described, starting with the immune cells and cytokines that act together in inflammation and remodeling. The goal is to aid researchers in understanding intimate connection between inflammation and remodeling of CRS and to offer novel ideas for future research.

## 1 Introduction

Chronic rhinosinusitis (CRS) is described as a chronic inflammatory disease of the mucosa of the nasal cavity and sinuses which can lead to 12 consecutive weeks of clinical symptoms such as nasal runny nose, nasal congestion, facial swelling, decreased sense of smell, headache and dizziness. In addition, signs of disease can be diagnosed by endoscopy or associated CT scan changes (1, 2).The pathology of CRS is commonly characterized by persistent mucosal inflammation and tissue remodeling (3). The immune response in CRS can be divided into three main inflammatory endotypes based on a distinctive characteristic spectrum which is composed of various inflammatory mediators, immune cells, and physiological functions (4–6). The three immunological subtypes display the following characteristics: Type 1 inflammation is primarily mediated by 1 helper T lymphocytes (TH1), type 1 innate lymphoid cells (ILC1), natural killer (NK) cells, and neutrophils, with a priority expression of interferon-γ (IFN-γ), tumor necrosis factor-α(TNF-α), and IL-12. Type 2 inflammation is predominantly driven by TH2, ILC2, eosinophils, macrophages, and B cells, and is associated with interleukin-4 (IL-4), IL-5 and IL-13. Lastly, Type 3 inflammation involves neutrophils, TH17 as well as ILC3 and is characterized by elevated levels of IL-17 and IL-22. The terms Th1, Th2 and Th17 inflammation have been also used to describe these three endotypes. The remodeling features of CRS include goblet cells proliferation, basement membrane thickening (BMT), subepithelial edema and fibrosis, subepithelial glandular hyperplasia, collagen deposition, epithelial-mesenchymal transition (EMT),and osteitis (7–9). The typical remodeling related factors that can regulate these features are matrix metalloproteinase (MMP), tissue inhibitor of metalloproteinases (TIMP), transforming growth factor-β (TGF-β), vascular endothelial growth factor (VEGF), platelet-derived factor (PDGF), bone morphogenetic protein (BMP) and osteopontin factors (10–12). Interestingly, at the same time, it has been reported that the two most common phenotypes, chronic rhinosinusitis with nasal polyps (CRSwNP) as well as chronic rhinosinusitis without nasal polyps(CRSsNP), possess different immunomodulatory mechanisms and remodeling characteristics (2, 13). However, the potential link between inflammation and remodeling in CRS is not well understood as for a very long time, remodeling characteristics in CRS were thought to be secondary processes resulting from the protracted inflammatory process (14). But, the findings reported in early CRS studies that increased fibroblast and collagen deposition in the nasal cavity can occurs prior to local inflammation has opened new areas of investigation (15).

The pathogenesis of CRS is complex as it is a chronic disease that requires long-term maintenance treatment to control the condition (16). In recent years, control has been proposed as a more subtle outcome indicator by different experts, which refers to the maintenance of disease performance at an acceptable level (17). At present, the dominant methods of control include pharmacological treatments, followed by surgical intervention. However, with advent of refractory CRSwNP that persists or recurs despite long-term pharmacological and surgical treatment and acute exacerbations of chronic rhinosinusitis (AECRS) that can cause transient worsening of symptom intensity in patients with CRS, the traditional methods of medication and surgery do not often provide ideal results. Additionally, inspired by significant advances in asthma treatment, biologics have become the focus of CRS treatment in recent years (**Table 1**). In fact, some biologics have shown remarkable improvements in the severity of refractory CRSwNP and AECRS on both the subjective and objective indicators (18, 19). These biologics, primarily focusing on cytokines, cytokine receptors or antibody bindings, appear to provide a new perspective that the different cytokines, immune cells and remodeling factors related to CRS could potentially be key in breaking the prevailing belief that remodeling and inflammation do not interact during disease progression. Thus, based on these recent findings in the field, the current article reviews the potential roles of cytokines, immune cells and remodeling factors in the inflammation and remodeling mechanisms of CRS. The goal is to further explore the possible relationship between inflammation and remodeling, thus laying a theoretical foundation for the formulation of more refined and individualized CRS treatment strategies in the future.

**Table 1:** Biological agents approved or in clinical trials for CRSwNP.

References	Biologic	Target	Development Status for CRS
Bachert C et al, 2019 (1)	Dupilumab	IL-4Rα	EMA and FDA approved for CRSwNP, Phase 3 trials concluded
Gevaert P et al, 2020 (2)	Omalizumab	IgE	EMA and FDA approved for CRSwNP, Phase 3 trials concluded
Jk H et al, 2021 (3)	Mepolizumab	IL-5	EMA and FDA approved for CRSwNP, Phase 3 trials concluded
Bachert C et al, 2022 (4)	Benralizumab	IL-5Rα	Phase 3 trials concluded
ClinicalTrials: NCT02799446	Reslizumab	IL-5	Under phase 3 trial
ClinicalTrials: NCT05281523	Depemokimab	IL-5	Under phase 3 trial
ClinicalTrials: NCTO4851964	Tezepelumab	TSLP	Under phase 3 trial
ClinicalTrials: NCTO3614923	Etokimab	IL-33	Under phase 2 trial

IL, interleukin; EMA, european medicines agency; FDA, food and drug administration; IgE, immunoglobulin E; TSLP, thymic stromal lymphopoietin.

## 2 Important cytokines involved in CRS remodeling and inflammation

It has been established that cytokine expression and regulation serve as the most crucial factors in the pathological mechanisms of CRS that have been identified after years of research development. The TH1, TH2, TH17 and remodeling-associated cytokines that can participate in CRS inflammation and remodeling (**Table 2**), as well as the pathways of cytokine action, have been discussed in the sections that follow.

**Table 2:** Cytokines Influencing Remodeling and Inflammation.

Effectors Targets	Tumor necrosis factor-α	Interferon-γ	Interleukin-4/ Interleukin-13	Interleukin-5	Interleukin-17	Transforming growth factor-β	Fibroblast growth factors
Neutrophils	+	+	/	/	/	/	/
Eosinophils	+	/	/	+	/	—	/
Macrophages	+	/	/	/	/	/	+
Fibroblasts	/	—	/	/	+	+	+
Fibrosis	—	—	+	/	+	+	+
Mucosal edema	/	/	+	+	/	+	/
Epithelial mesenchymal transition	+	/	+	/	/	+	/
Nasal polyps	/	/	+	+	+	/	/

'+' indicates promotion, '–' indicates inhibition, '/' denotes not mentioned in the text.

### 2.1 Impact of cytokines of the TH1 on remodeling and inflammation

The TH1 cytokines involved in CRS are mainly TNF-α and IFN-γ which are produced by Th1 cells, cytotoxic T cells, NK cells as well as ILC1 and prior studies have reported that both TNF-α and IFN-γ play a substantial role in the development of inflammation and remodeling pathology, where the inflammatory response primarily driven by TNF-α and IFN-γ is even considered to be the main characteristic of TH1 inflammation (20).

It has been found that multiple pathways can be used by TNF-α to have an impact on inflammation and remodeling. TNF-α is frequently regarded as a key pro-inflammatory factor in the pathogenesis of CRS due to the fact that nasal polyp tissues have been observed to express markedly high levels of TNF-α (21). In fact, previous studies have revealed that TNF-α can directly harm the nasal mucosal epithelium by causing mononuclear-macrophages formation, triggering their migration, and increasing their cytotoxicity. It can also activate the T lymphocytes, stimulate the production of immunoglobulin by B lymphocytes (22), and increase neutrophil levels, by promoting eosinophil survival, and enhancing their cytotoxic effects (23). In CRSsNP, which constitutes up to more than two-thirds of CRS cases (24), fibrosis has been identified as the primary remodeling feature (25). This condition is typically characterized by excessive fibroblast proliferation and deposition of the collagen-rich extracellular matrix (26). TNF-α, on the other hand, has been demonstrated to exert significant antifibrotic properties in preclinical models of TNF receptor-deficient mice (27), and in experiments with TNF-α blockers alone (28). EMT is a process that can effectively convert polar epithelial cells into cells with a mesenchymal phenotype (29) and it facilitates a pervasive inflammatory damage repair and remodeling process that takes place in mucosal and skin barriers. It is well-known that TNF-α acts as a key signal for EMT induction in the tumor environment (30), and it has the ability to promote accelerated EMT through a variety of mechanistic pathways to increase the invasiveness of cancer cells (31, 32). Additionally, increased TNF-α expression in CRS can lead to increased EMT, which could be potentially related to the development of subepithelial fibrous tissue as well as thickening of the basement membrane and thickening of nasal sinus mucosal tissue on sinus CT images (29).

IFN-γ is a key cytokine that predominantly aids in host defense againstTh1 inflammation caused by intracellular pathogens (33), but it has also been reported to display strong antagonistic effects in a number of fibrotic disease models. IFN-γ plays a complex role in regulation of inflammation in CRS. On the one hand, it can promote inflammation by causing neutrophil oxidation, phagocytosis, as well as chemotaxis, disrupting tight junction proteins, and inducing apoptosis in nasal mucosal epithelial cells (34). On the other hand, it can protect by preventing the development of more pathogenic T cell phenotypes (Th2 and Th17). The impact of IFN-γ on remodeling is reflected in its prominent antiproliferative and antifibrotic effects (35) and IFN-γ has been found to reduce fibrosis in liver (36) and kidney (37) fibrosis models by inhibiting TGF-β activity, or can also inhibit fibroblast activation and proliferation to exert anti-fibrotic effects (37) and suppress collagen synthesis (38). Therefore, it is highly likely that the elevated levels of IFN-γ in CRS can also affect the remodeling of the nasal mucosa’s fibrosis.

### 2.2 The impact of TH2 cytokines on remodeling and inflammation

Eosinophil and mast cell infiltration, goblet cells proliferation, elevated immunoglobulin(Ig) E levels, and the production of cytokines like IL-4, IL-5, and IL-13 by ILC2, T cells2, and Th2 cells have all been identified as the characteristics of the TH2 immune response in CRS (39). It has been reported that different CRS phenotypes have TH2 immune responses, and medium CRSwNP can have up to 80% of them (40). There are even several guidelines published for the management of differentiated CRS with type 2 and non-type 2 immune responses. The TH2 immune response can be found in the different TH2 inflammatory factors such as IL-4, IL-5, and IL-13 which have been extensively studied in CRS due to their association with more severe symptoms, higher recurrence rates, and more complex concomitant symptoms. They can be identified as biomarkers in the peripheral blood and nasal mucosal tissue of CRS patients, and have also been shown to play a significant role in the pathological mechanisms of both inflammation and remodeling in CRS (41).

A pair of closely related cytokines, IL-4 and IL-13, share the IL-4R/signal transduction and activator of transcription 6 (STAT6) signaling pathway for mediating their actions (42). Inflammation and remodeling in response to CRS also reveal similarities between IL-4 and IL-13 pathological manifestations. For instance, IL-4 can exert immunomodulatory effects on B cells, T cells, mast cells, as well as macrophages, and serves as an autocrine growth factor for helper T cells that can influence CRS inflammation. In CRS, IL-4 can stimulate B cells to differentiate into the plasma cells and produce IgE that can bind to the mast cells and cause the release of a number of inflammatory mediators, particularly eosinophil chemokines, which ultimately can damages sinus mucosal tissues and promote the development of nasal polyps (43). Moreover, IL-4 can induce the expression of vascular cell adhesion factors by endothelial cells, and it can also facilitate the binding of monocytes, lymphocytes, as well as eosinophils to the vascular endothelial adhesion, which ultimately can augment the overall inflammatory response (44). IL-13, on the other hand, is a pleiotropic cytokine that is primarily produced by activated Th2 cells, and prior studies have shown that the nasal mucosal tissues from CRS patients have significantly elevated levels of IL-13 (45). IL-13 is involved in the nasal mucosa’s defense mechanism against the respiratory viral and bacterial infections and it can activate B cells to regulate IgE production and epithelial remodeling (46, 47). It can also act synergistically with other proinflammatory cytokines. As a result of the discovery that IL-4 and IL-13 play a critical role in the inflammatory process that can lead CRS, several biological agents that target the IL-4/IL-13 pathway have been discovered for use in CRS. Although IL-4 and IL-13 can influence CRS remodeling in remarkably identical fashion, IL-13 generally has been reported to have a greater impact. It is believed that IL-13 is a crucial component in pro-fibrosis because numerous studies have indicated that it can either directly or indirectly promote fibrosis (48, 49). It had been previously also hypothesized that IL-4 could be responsible for the pro-fibrotic effect of IL-13 (50). However, it has been found that IL-13 can still exert its fibrosis-inducing effect when the traditional IL-4R/STAT6-mediated signaling pathway has been blocked, i.e., IL-13 can induce fibrosis by activating additional signaling mechanisms through its own receptors (51). In addition, through activating its downstream signaling pathways, IL-13 can also stimulate TGF-β expression, which can enhance fibroblast activation and collagen deposition, thereby causing inflammatory edema and thickening of the basement membrane (52). Moreover, IL-13 and IL-4 can work in conjunction to suppress the tissue fibrinogen activator expression, activate coagulation factor XIIIa, and negatively modulate eosinophil levels. These events can result in increased fibrin deposition and cross-linking, tight tetrameric complex formation, and worsened edema remodeling in rhinitis (53).

As IL-5 has been identified a crucial component in eosinophil proliferation, chemotaxis, differentiation, activation, and survival (54), it appears that IL-5 can primarily affect inflammation and remodeling through eosinophils during the pathological development of CRS. For example, in CRS, activated IL-5 can promote a large number of eosinophils to migrate into the mucosa and strengthen their adhesion, thus promoting nasal mucosal inflammation, which has been established as the tissue basis of nasal polyps (55). The expression of IL-5, on the other hand, was reported to be significantly increased in CRS, both in the nasal mucosal tissue and serum, and it was closely correlated with both the subjective and objective measures of the severity of the disease (56). Eosinophils play a vital part in how IL-5 can affect remodeling as well, and hence the topic is discussed in more detail under eosinophils below.

In conclusion, TH2 cytokines IL-4, IL-5, and IL-13 not only contribute to CRS inflammation by rupturing the epithelial barrier, mediating the cilia dysfunction and mucus production, mediating altered nasal mucosal macrophage function, reducing innate immune function in the nasal cavity stimulating the development of mucosal edema and pseudocysts (57), but can also display strong pro-fibrotic properties. Overall, they possess potent pro-fibrotic properties in the context of CRS remodeling, which are intimately linked to the onset of fibrosis (58).

### 2.3 The impact of TH17 cytokines on remodeling and inflammation

ILC3 and Th17 cells can all produce two main cytokines of the TH17 immune response, IL-17, and IL-22. An important characteristic of the TH17immune response is inflammation that is mainly driven by IL-17 and IL-22 cytokines (59). A number of related to CRS have also shown that IL-17 and IL-22 can have a significant impact on the inflammatory process, but their influence on CRS remodeling is currently unknown. IL-17 is a general term for the various cytokines like IL-17A, B, C, D, E, and F (60), among which abnormal expression of IL-17A has been strongly associated with chronic inflammation and autoimmune diseases (61)and it has been reported to play a significant role in promoting nasal polyp formation (60). Thus, by influencing the pro-inflammatory response of inflammatory cells in the nasal mucosa, high expression of the IL-17 protein found in CRS patients could effectively contribute to the pathogenesis of nasal polyps (60). Additionally, since IL-17 was discovered to be significantly pro-fibrotic in skin (62), liver (63), lung (64), intestine (65), kidney (66), and heart (67)models of fibrosis, IL-17 is also identified to function as a significant pro-fibrotic factor. For instance, in asthma model mice, anti-IL-17 treatment was found to decrease the lung inflammation, edema, oxidative stress, and extracellular matrix remodeling (68). It could also induce collagen production in myofibroblasts and modulate the expression of MMP-3, MMP-9, and TIMP1 (69, 70). Therefore, it is implied that IL-17 may also be crucial for the remodeling of the nasal mucosa in CRS.

A member of the IL-10 cytokine family, IL-22 has been reported to be crucial for mucosal intrinsic immunity in the respiratory, digestive, and skin tracts, because it can aid in anti-microbial defense, the protection and repair of the tissue damage, and acute phase responses (71). Interestingly, prior studies on IL-22 in CRS, however, are still unclear, and the inconsistent results of IL-22 content measurements in CRS could be a significant impediment. This could be attributed to the fact that nasal polyps themselves have more pathological types and can be affected by various factors, such as the site of sampling and the application of hormonal drugs at the time of the study, as well as the different ethnicities of the study subjects (72). However, in terms of influencing the remodeling, researchers have hypothesized that its function is similar to that of IL-10; however, in contrast to the hazily predicted remodeling effects, inflammatory effects of IL22 are relatively evident in CRS, where it was discovered to exhibit a favorable effect on the expression of different adhesion molecules and chemokines. Thus, through affecting these important functions, it can attract diverse inflammatory cells, which in turn can intensify the Th2-type immune response and ultimately induce pathological effects (73).

### 2.4 The impact of remodeling-related cytokines on remodeling and inflammation

Normally considered to be typical cytokines affecting CRS remodeling, TGF-β, fibroblast growth factors (FGF) and VEGF have been found to have a profound impact on the pathological process of CRS inflammation through prior research.

TGF-β refers to TGF-β1 when not otherwise specified because TGF-β1 has an overwhelming predominance of up to 80% and is the most abundant member of TGF-β family. Given that TGF-β1 is the most fibrogenic factor (74), it has been proposed that the differences in TGF-β levels between CRSwNP and CRSsNP could contribute to the different remodeling characteristics of CRS tissue remodeling (75). Moreover, When it comes to its inflammatory impact on CRS, TGF-β1 has been reported to have both pro- and anti-inflammatory effects. For example, studies have elegantly shown that TGF-β1 can promote fibrosis through a variety of pathways as CRS pathology develops.

The number of fibroblasts is directly correlated with the expression of TGF-β1, which has been found to induce fibroblast proliferation and differentiation into the myofibroblasts (76). Additionally, TGF-β1 can regulate the balance between TIMP and MMP, mediate collagen release and extracellular matrix synthesis (77). Furthermore, TGF-β is the most effective inducer of EMT (78) and can contribute significantly to EMT through modulating Smad signaling (79). In conclusion, TGF-β can mediate EMT and induces extracellular matrix (ECM) protein expression in mesenchymal cells by regulating the stromal MMP and TIMP expression, thus leading to increased stromal permeability, ECM degradation, albumin deposition, and nasal mucosal edema. It can thus actively participate in the tissue remodeling of the sinus mucosa in CRS. TGF-β1, meanwhile, also plays a dual role in the inflammatory immune response in CRS. On the one hand, TGF-β1 can increase inflammation by triggering the differentiation of T lymphocytes into inflammatory Th17 (80), but on the other hand, it can suppress the production of various inflammatory cytokines and mediators (e.g. IL-1, IL-8, TNF-α) (81), thus suppressing the release of mediators from eosinophils and increasing their apoptosis rate (82). It can also significantly inhibit the proliferation and cytokine production of naive T cells as well as Th1 as well as Th2 clones (83), and promote the differentiation of T regulatory cell (84) by exerting diverse anti-inflammatory effects.

FGF is a crucial cytokine involved in the various developmental processes like cell proliferation, differentiation, and migration as well as in injury and tissue remodeling (85). FGF can promote fibrosis when it is involved in CRS remodeling by transforming the epithelial cells into fibroblast-like cells (86), whereas it was discovered that significantly more basic FGF-2 was found in the nasal secretions of CRS patients (87), which can attract leukocytes to secrete the different inflammatory mediators in acute and chronic inflammatory conditions (88). FGF-2 can also stimulate the development of inflammatory cells and improve their infiltration into tissues, including the macrophages and T lymphocytes (89). Additionally, research findings using FGF receptor inhibitors to block FGF signaling have revealed that it can cause inflammatory reactions that involve TNF-α (90).

An important factor regulating the tissue angiogenesis, tissue proliferation remodeling, and increasing vascular permeability is VEGF, which is a mitogenic peptide that is specific to endothelial cells (91–94). At the beginning of CRS, the nasal mucosal tissue is already neovascularized and as the proliferating tissue grows, the rate of VEGF positive expression increases substantially (95). Consequently, it is also believed that VEGF can control both the vascular growth and inflammation during CRS.

## 3 Important immune and effector cells involved in CRS remodeling and inflammation

The pathological mechanisms of CRS inflammation and remodeling are complicated due to the impact of the main immune and effector cells involved in this condition (**Table 3**). This can be attributed to the fact that these cells are able to exert differential effects on them at various stages of the disease development and because they also constitute a part of interconnected pathological networks that can sometimes have completely divergent effects on one another depending on the situation.

**Table 3:** Cells Influencing Remodeling and Inflammation.

Effectors Targets	Neutrophils	Eosinophils	Macrophages	Fibroblasts
Interleukin	+	+	+	+
Interferon-γ	+	/	/	/
Tumor necrosis factor-α	+	/	+	/
Fibrosis	+	+	+	+
Transforming growth factor-β	+	+	+	/
Mucosal edema	—	+	/	/
Nasal polyps	+	+	/	+
Epithelial-mesenchymal transition	+	+	/	/

Please refer to **Table 2** for the definitions of the special symbols used in this table.

### 3.1 Neutrophils

Neutrophils have been reported to play a significant role in the development of all forms of CRS pathology, both in terms of inflammation as well as remodeling, and their importance cannot be underestimated. Neutrophils are the primary effector cells of the intrinsic immune system and they exhibit a variety of synergistic functions that work together to eliminate pathogens (96). On the contrary, it was discovered that chronic inflammatory diseases associated with the respiratory system have persistent neutrophil infiltration, and the level of infiltration was positively correlated with the degree of inflammation and disease (97). Neutrophils can contribute to the immune response by secreting various cytokines, such as IL-36 and IL-33, IL-1, IL-6, IL-8, IFN-γ and TNF-α, to upregulate the inflammatory response, which is one of the major event in CRS (98). Additionally, CRSsNPs that clearly demonstrate the neutrophil infiltration, in addition to neutrophils that may contribute to nasal polypogenesis have been identified in non-eosinophilic CRSwNPs (99).

Neutrophils can display a variety of effects on CRS remodeling, including fibrosis, edema, EMT and cause an imbalance between MMP and TIMP. A number of studies have reported a positive correlation between fibrosis and neutrophils in CRS, probably because neutrophils are the primary source of TGF-β2-positive cells that can positively correlate with the myofibroblast number and fibronectin expression levels (100), i.e., the number of neutrophils which can positively correlate with the expression of pro-fibrotic factors, thus suggesting that neutrophils likely promote CRS via TGF-β2 in tissue fibrosis. The quantity of the neutrophils was also discovered to be negatively correlated with the level of edema (100). Neutrophil elastase, a serine protease released from the neutrophils has been found to affect goblet cells proliferation and mucin overproduction in patients with CRS (101, 102), EMT is a phenomenon involved in tissue remodeling that can eventually result in the local pools of fibroblasts, abnormal extracellular matrix deposition and the formation of nasal polyps (9). Whereas patients with neutrophil-dominated CRS can promote EMT via the IFN-γ pathway (103, 104); In addition, hypoxia-inducible factor-1α, which can induce EMT (105) has also been positively correlated with the number of neutrophils (100) and one of the primary mechanisms of pathological tissue remodeling in CRS is the imbalance between MMP and TIMP (7), where MMP-9 mainly degrades the gelatin, proteoglycan and elastin, a key factor in remodeling, whereas there is an association found between MMP-9 and neutrophils (106), which is an important source of MMP-9 (107).

### 3.2 Eosinophils

Eosinophils, which are a crucial part of leukocytes and like other granulocytes, are derived from the hematopoietic stem cells in the bone marrow can release granular materials that can damage tissue and advance inflammation (108). According to the prior reports, eosinophils have been implicated in a variety of biological functions (109), including mediating inflammation or inducing immunity. Moreover, studies have found that eosinophilia is positively correlated with the severity of CRS, thus indicating that more the number of eosinophils, greater is the severity of the disease (110). This is in addition to the pro-inflammatory role of eosinophils in releasing IL-4, IL-5, and IL-13 cytokines. Similarly, tissue eosinophilia has been significantly linked to the worsening of symptoms, lower quality of life, and substantially higher risk of recurrence in patients with CRSwNP after endoscopic nasal surgery (ESS) (111, 112). Hence, for these reasons, eosinophils are regarded as potential biomarkers for more severe and refractory diseases (113).

Eosinophils exhibit an extraordinarily strong positive correlation with various remodeling-related factors during the progression of CRS pathology, in addition to their capacity to release different pro-fibrotic and pro-angiogenic substances like TGF-β1, FGF-9, and VEGF. Interestingly, researchers have speculated that there exists a very close link between eosinophils and remodeling because sinus mucosal remodeling is more pronounced in CRS patients with co-morbid asthma and higher eosinophil load (14, 114, 115). The ability of eosinophil products to induce submucosal edema and epithelial damage has been highlighted in particular by the positive correlation between eosinophil cationic protein levels and edema (116). Additionally, BMT has been positively correlated with tissue eosinophil infiltration (117), but both edema and collagen deposition in the basement membrane in CRS have been positively correlated with eosinophil (100, 118), where increased albumin deposition mainly caused by eosinophil-driven inflammation (119). The ability of eosinophils to promote fibrosis may be attributed to TGF-β, whose main source is eosinophils (120), which can induce fibroblast proliferation and differentiation into the myofibroblasts, as well as possesses capacity to produce IL-11 and IL-17, which have significant pro-fibrotic effects (121). Furthermore, it has been reported that eosinophils and fibronectin levels can be significantly correlated in nasal polyps of CRS patients (122). Moreover, a stromal cell protein called periostein has been linked to extracellular matrix buildup and fibrosis in the tissue remodeling (123, 124). Interestingly, expression of this protein in the sinus tissues has been positively correlated with eosinophil infiltration (125). Eosinophil infiltration and EMT in CRS have also been linked, and it was found that eosinophils play a significant role in promoting EMT process (9). In addition, eosinophils can effectively contribute to the secretion of MMP-9 from the nasal mucosal epithelium and have been positively correlated with it (126, 127). Eosinophils are crucial for osteitis bone remodeling as observed by the positive correlation between eosinophilic inflammation and upregulated growth differentiation factors as well as exostein glycosyltransferases in osteitis of CRS patients (128).

Eosinophils undoubtedly play a significant role in regulating both mucosal inflammation and remodeling in CRS, but consistent remodeling was also observed in nasal polyps in Western and Asian populations in comparison to Asian patients with different inflammatory features and generally lower eosinophil levels (7), thereby presumably indicating that eosinophils may not be the primary factors influencing remodeling (129).

### 3.3 Macrophages

Macrophages have been recognized to play a key role in the development and resolution of inflammation (130). After phagocytosing cellular debris, invaders, neutrophils, and other apoptotic cells that appear after the tissue injury (131, 132), local tissue macrophages and recruited monocytes quickly transform from M1-type macrophages, which secrete a wide variety of proinflammatory cytokines such as IL-1, IL-6, TNF-α, IL-17A and IL-12 (133, 134), to M2-type macrophages with an increased capacity for anti-inflammatory response. M2-type macrophages can contribute to the remodeling by secreting diverse cytokines like TGF-β1, IL-6, and IL-13 to promote the fibroblast survival, proliferation, myofibroblast activation, collagen production, and increased transcription of pro-fibrotic genes (135–138), as well as by secreting factors like IL-10 and TGF-β1 which can exhibit anti-inflammatory effects (139). Moreover, both M1 and M2 macrophage numbers have been reported to increase during the pathological progression of CRS, with an especially large increase in M2 macrophage numbers in the later stages (140). This finding suggests that macrophages also exhibit a pattern from M1 to M2 types, or from pro-inflammatory to anti-inflammatory, during this process (141).

Given the intricate relationship between the reported pleiotropic effects of macrophages on inflammation and tissue remodeling in CRS, it has been hypothesized that the development of nasal polyps could be closely linked to the tissue remodeling caused by M2 macrophage polarization (142). It has been established that because macrophages can serve as a significant cellular source of different chemokines like eosinophil chemokines in the nasal mucosa (143), Th2 cytokines have been positively correlated with increased macrophage numbers during the pro-inflammatory developmental phase of CRS (140) and can directly or indirectly induce the production of Th2 cytokines thereby regulating the immune environment. In addition, M2 macrophages in CRS can significantly alter the vascular permeability (77), regulate associated inflammatory factors and control coagulation mechanisms (144), which can result in tissue remodeling in CRS. In conclusion, research has shown that monocytes-macrophages, depending on their polarization within the tissue as well as the type and stage of the disease, can play a complex but significant role in the regulation of inflammation, proliferation, and fibrosis (58).

### 3.4 T cells and innate lymphoid cells

T cells play multifaceted roles in regulating various physiological processes, which include recruiting effector cells, neutralizing infected cells, aiding B cells in immunoglobulin production and functioning as potential memory cells in innate immune system (145). Predominantly, T cells can be categorized into CD4+ T helper cells and CD8+ cytotoxic T cells. CD4+ T cells can further differentiate into five primary subsets: Th1, Th2, Th17, follicular helper T cells, and T regulatory cell (146). Th1 once activated by phagocytosed microbes along with support from ILC1s, can release IFN-γ, TNF-α, and TNF-β. These cytokines can then aid in the phagocytosis of the microbiome by activating macrophages and promoting antigen presentation, in addition to stimulating IgG production by B cells, neutrophils, and inducing local tissue inflammation (147). Th2 are primarily activated by the different parasites, which can trigger eosinophils, mast cells, ILC2s and enhance IgE production (148). Th2 can secrete IL-4, IL-5, and IL-13, thus contributing to the activation of eosinophils, mucus production and macrophage stimulation (149), thereby potentially leading to the production of several growth factors that can initiate tissue repair mechanisms. The Th17 subpopulation can activate neutrophils and monocytes to stimulate the secretion of IL-17A, IL-17F, and IL-22 (2). Most of T cells accomplish their objectives by producing a wide array of cytokines, which have been discussed in detail above in part two of this article.

Innate lymphoid cells (ILCs) are a subpopulation of innate immune cells that can produce a variety of cytokines that are compatible with the Th subpopulation of adaptive immune cells. These cytokines have been found to be crucial for the coordination of innate and adaptive immune responses. ILCs are also referred to as intrinsic immune cells, which are a subset of lymphocytes that are distinct from T and B cells and are primarily found in the mucosal barrier tissues. ILCs play a vital role in inflammatory diseases of the respiratory system by promoting lymphoid organ formation, enhancing immune responses and maintaining mucosal integrity (150). Moreover, based on the transcription factors and the cytokines produced, ILCs have been divided into three subtypes, ILC1s, ILC2s, and ILC3s (151), and these three cell subtypes produce high levels of the similar cytokines as TH cells, such as ILC1 secretes IFN-γ, ILC2 produces IL-5 and IL-13, and ILC3 secretes IL-17 as well as IL-22 (152), and thus play an important role in the pathogenesis of CRS.

### 3.5 Fibroblasts and goblet cells

The pathological development of CRS involves the epithelium, which can serve as the primary barrier by defending the host from external physical, chemical, and immune stimuli (153). Fibroblasts and goblet cells have been identified as significant contributors to the epithelium in this process. It has been reported that in comparison to CRSwNP, which exhibits more edema and less fibrosis, remodeling of CRSsNP can display a marked feature of BMT, fibrosis, and goblet cells proliferation (154). The contribution of fibroblasts and goblet cells to remodeling is undeniable, but more studies are needed to fully understand how inflammation can potentially modulate these cells.

Extracellular matrix that is rich in collagen is typically deposited concomitant with excessive fibroblast proliferation is considered as a symptom of fibrosis (26), and fibroblasts function as the primary effector cells that can promote fibrosis. For example, in organ related diseases like those of the lung, liver, and kidney, anti-fibrosis has been a hot topic of research. The complex mechanism of inflammation and remodeling of the nasal mucosa by fibroblasts in CRS has been thought to play an important role in the etiology and persistence of nasal polyps (155). However, because the proportion of fibroblasts in nasal polyps is significantly higher than that in the healthy nasal mucosa, in addition to promoting fibrosis, they can also release eochemokine, which plays an important role in stimulating eosinophil recruitment in nasal polyps (156), and also can mediate the release pro-inflammatory IL-6 and IL-8 cytokines (155). Therefore, it has been speculated that they may serve as the key source of inflammatory mediators (157).

The proliferation of goblet cells, a type of nasal mucosal epithelial cell whose primary function is to secrete plenty of mucus, can increase the secretion of mucin, the primary component of the mucus in the nasal membrane (158). Goblet cells are presence in abundance to secrete mucin in excess and hypersecretion of mucin is associated with pathological changes in CRS (159). In addition, increased mucus volume and viscosity arising as a result of hypersecretion by goblet cells can cause mucociliary dysfunction (160), mucus retention, and aggravation of inflammation (161). In addition, numerous proinflammatory cytokines have been demonstrated to control the goblet cells metaplasia and excessive mucin secretion (162), thereby creating a vicious cycle that can exacerbate the inflammatory response of CRS. As a result, some researchers have concluded that important pathogenic mechanisms of CRS include the proliferation and metaplasia of glandular cells and goblet cells as well as the promotion of sinusitis mucin expression (163).

## 4 Discussion

CRS is a highly heterogeneous disease with a relatively high prevalence. Its pathogenesis involves several factors, including microbial infection, immune dysfunction, sinus anatomy and concomitant diseases. The primary strategies employed for treating CRS are pharmacological drugs and surgical treatments, but given the complex heterogeneity of CRS, formulating universally applicable treatment modalities poses a significant challenge. All these factors inevitably lead us back to the core of CRS pathophysiology-inflammation and remodeling. In recent years, there has been growing interest in the area, yet surprisingly few reviews have explored this burgeoning field. Recently, Wang et al. (164) stratified CRS endotypes by amalgamating inflammatory biomarkers with typical remodeling factors, identified five clusters and subsequently classified them into endotypes: non-type 2 inflammation (clusters1 and 2) and type 2 inflammation (clusters 3, 4, and 5). However, like most of the previous studies in this field, this research has not delved deeply into the complex relationship reported between inflammation and remodeling. Moreover, relationship between inflammation and remodeling has been drawn from experiences related to asthma and other upper respiratory diseases, but there is no consensus in the academic community on whether CRS follows identical mechanisms and whether its internal remodeling is reversible. Additionally, while pharmacological drugs that are effective against CRS inflammation have been identified, their role in remodeling is not clearly defined. Therefore, this dearth of comprehensive reviews presents an opportunity for our article to fill a significant research gap and provide a fresh, novel perspective on inflammation and remodeling in CRS.

We have explored the complex roles of cytokines, immune cells and remodeling factors in CRS inflammation and remodeling in the present study, which led us to recognize that in the pathological setting of CRS, inflammation as well as remodeling are closely interrelated, and that they promote each other to form positive feedback thereby exacerbating the pathology. It is therefore reasonable to assume that inflammation and remodeling in CRS are in a dynamic equilibrium that is clearly interrelated. Although the interactions between immune cells and cytokines and their roles in different environments can make it difficult to comprehend this complex relationship, a clear understanding of their relationship as an important feature of CRS is of key importance for our in-depth interpretation of the pathological mechanisms of CRS. In addition, precision medicine which has been developed for the inflammatory and molecular features of CRS subtypes has been regarded as an important avenue for CRS research. The various biologics developed for targeting specific immune cells or cytokines have opened up the possibility of individualized and targeted therapy for CRS, leading the way for future research, although their general use is limited by high cost and insufficient evidence. Therefore, in future studies, one should utilize advanced technologies such as multi-omics analysis, single-cell RNA sequencing, and artificial intelligence analysis to completely elucidate of the role of different inflammatory cells, cytokines, and remodeling-associated factors on inflammation and remodeling, and thus promote the effectiveness and precision of the treatment.

## 5 Limitations

First, given the limited data available related to the relationship between inflammation and remodeling, the majority of literature covered in our study is relatively outdated, and may not completely represent current research findings and perspectives. Second, a large body of the evidence presented has not undergone meta-analysis, a type of aggregate statistical analysis that can facilitate the extraction and reanalysis of data from the multiple studies to obtain a more profound and conclusive understanding. In the absence of meta-analysis, the validity, consistency and reliability of the provided evidence may be a little hard to determine. Thus, although our study furnishes a plethora of evidence, the lack of meta-analysis might limit its overall utility. Finally, the sheer breadth of topics covered in our study could impose limitations on the depth and scope of the discussion. The detailed content segmentation might aid in better understanding and investigating a particular topic, but excessive subdivision could also render the research fragmented and disjointed, thereby potentially impinging on the integrity and coherence of the theory. Overall, these limitations could affect the reliability and validity of our study. Future directions in inflammation and remodeling research should aim to provide high-quality randomized controlled trials, which can lead to the creation of robust evidence-based guidelines to assist the clinicians.

## 6 Conclusions

Our preliminary research has elegantly unveiled the intricate interplay between inflammation and remodeling in CRS, where typical inflammatory cells and cytokines, along with the several remodeling-associated factors, exhibit a complex pattern of mutual enhancement or suppression, thus posing significant challenges for precision medicine. This necessitates more research related to different advanced technologies such as multi-omics analysis, single-cell RNA sequencing, and artificial intelligence analytics which can effectively aid to deepen our understanding of the relationship between inflammation and remodeling. This enhanced knowledge could provide novel strategies both for the prevention and personalized treatment of CRS.

## Author contributions

HL, TZ and XG selected the topic. XG and YF thought through idea and frame of the article. ZH, HF and XG collected the related studies. XG wrote the first draft of the manuscript. YW and YH created the tables. HL, TZ and YF proofed the text and tables. HL, TZ and YF revised the manuscript. All authors contributed to the article and approved the submitted version.
